# Assessment of the Efficacy and Safety of Early Intracoronary Nicorandil Administration in Patients With ST-Elevation Myocardial Infarction Undergoing Primary Percutaneous Coronary Intervention

**DOI:** 10.7759/cureus.25349

**Published:** 2022-05-26

**Authors:** Himanshu Gupta, Shishirendu Parihar, V.D. Tripathi

**Affiliations:** 1 Cardiology, Shyam Shah Medical College, Rewa, IND

**Keywords:** st-elevation myocardial infarction, percutaneous coronary intervention, no-reflow phenomenon, myocardial perfusion, morality

## Abstract

Background

No-reflow phenomenon (NRP) remains a challenge in ST-elevation myocardial infarction (STEMI) patients. We determined the efficacy and safety of early intracoronary administration of nicorandil as an adjunct to primary percutaneous coronary intervention (pPCI) in STEMI patients to reduce the risk of NRP.

Materials and methods

In this single-center case-control prospective study, 100 STEMI patients who underwent pPCI had thrombectomy performed using a suction catheter, and tirofiban (10 mg/kg) was injected distal to the vascular lesion. All patients were divided into two groups. Group A was a treatment group (nicorandil, n=50) and group B was a control group (placebo, n=50). The primary endpoint was the composite endpoint of in-hospital cardiovascular mortality or unscheduled re-hospitalization due to deterioration of congestive heart failure that was assessed with the help of brain natriuretic peptide (BNP), left ventricular end-diastolic diameter (LVEDD), and left ventricular ejection fraction at six months following pPCI. The secondary endpoints were thrombolysis in myocardial infarction (TIMI) flow grade, TIMI myocardial perfusion grade (TMPG), the incidence of reperfusion arrhythmias like ventricular tachycardia and ventricular fibrillation, and ST segment elevation resolution (STR) on ECG following pPCI.

Results

The in-hospital cardiovascular mortality and re-hospitalization rates were 2% and 6% in the nicorandil group, whereas it was 6% and 14% in the control group. On the 180^th^ day of admission, the nicorandil group had significantly lower values of brain natriuretic peptide (348.45±112.32 pg/ml vs. 541.11±152.68 pg/ml, p=0.021) and left ventricular end-diastolic diameter (54.12±3.56 mm vs. 60.62±4.98 mm, p=0.011) than the control group. Nicorandil group had a significantly higher number of patients who attained TIMI 3 (p=0.022), TMPG 3 (p=0.034), and STR (p=0.008) than the control group. Ventricular arrhythmia was significantly lower in the nicorandil group than in the control group at 24 hours following pPCI (p=0.012).

Conclusion

Early intracoronary administration of nicorandil during pPCI may decrease the occurrence of NRP, in-hospital cardiovascular mortality, and re-hospitalization rates, as well as improve coronary blood flow and reduce reperfusion arrhythmia in STEMI patients.

## Introduction

Early active initiation of successful revascularization via primary percutaneous coronary intervention (pPCI) is critical for the treatment of ST-elevation myocardial infarction (STEMI) [1]. However, reopening of an occluded coronary artery with the help of pPCI does not always result in optimal myocardial perfusion due to the occurrence of myocardial reperfusion injuries, such as reperfusion-associated arrhythmias, recurrent chest pain, and even no-reflow phenomenon (NRP), which occurs in 5% to 25% of the patients [2]. The NRP is induced by distal microthrombus embolization after balloon dilatation or stent insertion, as well as microvascular endothelial damage, thromboembolism, oxidative stress, microvascular spasm, and inflammation caused by ischemia-reperfusion injury [2,3]. Furthermore, it has been linked to greater infarct size, greater left ventricular contractile impairment, malignant arrhythmia, cardiac mortality, and poor prognosis [3]. Nowadays, numerous pharmacological therapies have been shown to be beneficial in reducing coronary microvascular impairment and blockage, as well as in avoiding NRP and major adverse cardiac events. Nicorandil is among the most effective medications for treating reperfusion injury [2].

Nicorandil is a combination of adenosine triphosphate (ATP)-sensitive channel opener and nitrate that improves the coronary microvascular impairment and blockage through dilatation of the small coronary arteries [4]. Various hypothesized reasons underlying nicorandil's cardioprotective action are anti-free radical and neutrophil-modulating characteristics, ATP-sensitive potassium channel opener, imitating ischemia preconditioning, and vasodilatation of small coronary and peripheral arteries [5]. However, there is a scarcity of data on nicorandil administration in Indian patients with STEMI. In light of this, the current study was conducted to assess the efficacy and safety of early intracoronary nicorandil administration as an adjuvant to pPCI in STEMI patients in order to reduce the risk of NRP.

## Materials and methods

This was a single-center case-control prospective study performed at the department of cardiology in the tertiary health care center from April 2021 to December 2021 to assess the safety and efficacy of early intracoronary administration of nicorandil to avoid the occurrence of NRP following pPCI in STEMI patients. The study included 100 patients who were segregated into two groups: group A was the treatment group (nicorandil, n=50), and group B was the control group (placebo, n=50). None of the patients were informed about the study groups. Each patient provided written informed consent. The study was approved by Institutional Ethics Committee and adhered to the tenets of the Declaration of Helsinki.

Included patients were aged >18-80 years with ST-elevation (≥0.1 mV elevation in ≥2 anatomically contiguous leads), had cardiac troponin I level above the normal range, without incidence of myocardial infarction (MI), percutaneous coronary intervention (PCI), or coronary artery bypass grafting, total coronary artery obstruction, with blood pressure >90/60 mm Hg, and arrived at the hospital within 12 hours of STEMI onset. Patients who had a history of MI, severe hypotension at the time of onset of STEMI symptoms, and kidney impairment (serum creatinine >2 mg/dL) were all ruled out from the study.

All the patients received 300 mg of aspirin and 180 mg of ticagrelor orally before pPCI. Local anesthesia was administered in both groups. Then, Seldinger coronary angiography was done in both groups through a radial approach. The lesion in the coronary artery was successfully crossed with Runthrough® NS guidewire (Terumo Europe NV, Belgium). Aspiration of the thrombus was done with the help of a Thrombuster® catheter (Kaneka Corp., Japan). In case of any obvious residual stenosis was found, the first step was to expand the balloon. After that, repeated suction catheter aspiration was performed. A dose of 10 µg/kg of tirofiban injection was administered into the distal part of the lesion. Then, patients were segregated into two groups: group A was the treatment group (nicorandil), and group B was the control group (placebo).

In regard to group A, a dose of 2 mg of nicorandil was administered distally to the coronary artery lesion with the help of a thrombus aspiration catheter. The administration of the drug was performed distally to the coronary artery lesion in order to prevent the low drug concentration in the thrombus-affected area. Angiography was performed again five minutes after receiving the nicorandil. Nicorandil was administered again when the thrombolysis in myocardial infarction (TIMI) flow grade of the affected coronary artery was found to be ˂3. All patients received a total dose of nicorandil of not ˃6 mg. A successive dose of nicorandil was given with at least five minutes intervals to minimize the risk of side effects.

In regard to group B, a 2 ml saline was injected distal to the thrombus using the thrombus-aspiration catheter. Angiography was performed again five minutes after receiving the saline injection. The saline injection was given again when the TIMI flow grade of the affected coronary artery was found to be ˂3. All patients received a total dose of saline of not ˃6 ml. The suction catheter was used to deliver 100-200 µg sodium nitroprusside distally to the site of the vascular lesion when TIMI flow grade was found between 0-2. There were no outside surgeons involved in any of the procedures.

The primary endpoint of the study was the rate of in-hospital cardiovascular mortality or unscheduled re-hospitalization due to the deterioration of congestive heart failure (CHF) that was assessed with the help of brain natriuretic peptide (BNP), left ventricular end-diastolic diameter (LVEDD), and left ventricular ejection fraction (LVEF) at six-month following pPCI.

Increased exercise-induced dyspnoea, nocturnal dyspnoea, orthopnoea, and the development of radiological symptoms of heart failure were all symptoms of worsening heart failure. If these symptoms were present, the patient was admitted to the hospital and was given diuretics intravenously.

The secondary endpoints of the study were assessed immediately following reperfusion. These endpoints included the following: TIMI flow grade, TIMI myocardial perfusion grade (TMPG), the incidence of reperfusion arrhythmias like ventricular tachycardia and ventricular fibrillation, and ST segment elevation resolution (characterized as a reduction of >50% in ST elevation) on electrocardiogram (ECG) following pPCI.

The TIMI flow grade of 0-2 following PCI, prior to coronary angiography, following stenting, and at the completion of the surgery, in the absence of residual stenosis, coronary artery dissection, thromboembolism, vasoconstriction, or any other mechanical impediments was termed as a no-reflow phenomenon.

An ECG was taken pre- and post-PCI. We used the 12-lead ECG's entire sum of ST segment elevation from the J point to the point attained 20 ms later to assess the resolution of ST elevation. The entire sum of the ST elevation in leads V1-6, I, and augmented vector left (aVL) was used to assess anterior infarction and the entire sum of the ST elevation in leads II, III. The augmented vector foot (aVF) was used to determine the presence of non-anterior infarction. The ST resolution (STR) was assessed by a trained physician who was unaware of the patient group allocation. To compute the sum of the ST segment deviations (ΣST), the sum of ST segment depressions recorded in reciprocal leads was added to the sum of ST segment elevations by using the following formula: [ΣST (admission) - ΣST (after PCI)]/ ΣST (admission). Patients were segregated into two groups according to ST segment deviation: a) patients who had a 50% reduction in ST segment deviation, and b) patients who had not a 50% reduction in ST segment deviation.

The ECG monitoring in the cardiac catheterization unit and the coronary care unit was used to determine the types and frequencies of ventricular arrhythmias. Malignant ventricular arrhythmia was characterized as ≥3 successive complexes originating in the ventricles at a heart rate of ˃100 beats per minute or ventricular fibrillation within 24 hours following angioplasty.

Follow-up was done at one, three, and six months via outpatient clinic visits and telephone calls to monitor the changes in the symptoms, the medications taken, and re-hospitalizations due to the deterioration of CHF.

Categorical data are presented as numbers and percentages, and continuous data as mean ± standard deviation (SD). A comparison of continuous data between the groups was performed by using an independent sample t-test. The four-fold contingency table χ^2^ test was used to compare categorical variables between the two groups. A p-value of <0.05 was regarded as statistically significant. Statistical tests were executed by using the SPSS statistical software, version 25.0 (IBM Inc., Armonk, USA).

## Results

Out of 100 patients, 50 patients were included in the nicorandil group, and the rest of the 50 patients was included in the control group. The mean age of the patients in the nicorandil group and control group was 68.32±3.24 and 67.34±4.23 years, respectively. The two groups were similar regarding baseline characteristics (Table 1).

**Table 1:** Baseline characteristics of study patients among nicorandil and control groups Data is presented as n (%) and mean ± SD. *All of the medications were intended for long-term usage.

Characteristics	Nicorandil (n=50)	Control (n=50)	p-value
Age, years	68.32±3.24	67.34±4.23	0.899
Male	35 (70%)	36 (72%)	0.784
Hypertension	28 (56%)	30 (60%)	0.782
Low-density lipoprotein cholesterol level >70 mg/Dl	17 (34%)	15 (30%)	0.447
Type 2 diabetes	21 (42%)	18 (36%)	0.482
Smoking history within one year	23 (46%)	25 (50%)	0.742
Stent length, mm	20.23± 6.82	20.48± 6.17	0.456
Medications before primary percutaneous coronary intervention*			
Aspirin	50 (100%)	50 (100%)	-
Ticagrelor	50 (100%)	50 (100%)	-
Medications during primary percutaneous coronary intervention*			
Tirofiban	50 (100%)	50 (100%)	-
Medications after primary percutaneous coronary intervention*			
Nitrate	45 (90%)	46 (92%)	0.682
Statin	49 (98%)	48 (96%)	0.782
Angiotensin-converting enzyme inhibitor	26 (52%)	28 (56%)	0.668
Angiotensin-receptor blocker	10 (20%)	9 (18%)	0.562
β-blocker	29 (58%)	28 (56%)	0.781

The values of concentrations in BNP levels, LVEDD, and left ventricular ejection fraction were identical in both groups on the first day of admission, whereas on the 180^th^ day of admission, the nicorandil group had significantly lower values of BNP (348.45±112.32 pg/ml vs. 541.11±152.68 pg/ml, p=0.021) and LVEDD (54.12±3.56 mm vs. 60.62±4.98 mm, p=0.011) than the control group (Table 2).

**Table 2:** Comparison of the presence of echocardiogram and brain natriuretic peptide among nicorandil and control groups Data is presented as mean ± SD.

	Day 1 after admission			Day 180 after admission		
	Nicorandil (n=50)	Control (n=50)	p-value	Nicorandil (n=50)	Control (n=50)	p-value
Brain natriuretic peptide, pg/ml	234.24±46.21	232.42±67.54	0.682	348.45±112.32	541.11±152.68	0.021
Left ventricular end-diastolic diameter, mm	48.43±1.48	49.14±2.34	0.832	54.12±3.56	60.62±4.98	0.011
Left ventricular ejection fraction, %	46.22±2.34	47.45±2.54	0.421	41.22±3.46	39.66±4.67	0.345

At a six-month follow-up, three (6%) patients in the nicorandil group and seven (14%) patients in the control group were re-hospitalized on account of worsening CHF. The in-hospital mortality rate due to cardiovascular causes was 2% in the nicorandil group, while it was 6% in the control group.

All patients had a TIMI flow grade of 0 at the commencement of the study. The nicorandil group performed considerably better and had a higher number of patients who achieved TIMI 3 than the control group both immediately after stenting (p=0.021) and at the end of the procedure (p=0.034; Table 3). 

**Table 3:** Comparison of thrombolysis in myocardial infarction flow grade of the affected vessel among nicorandil and control groups Data is presented as n (%).

Thrombolysis in myocardial infarction flow grade	Nicorandil (n=50)	Control (n=50)	p-value
Immediately after stenting			0.021
Grade 0	2 (4%)	4 (8%)	
Grade 1	2 (4%)	5 (10%)	
Grade 0-2	6 (12%)	14 (28%)	
Grade 3	37 (74%)	21 (42%)	
End of procedure			0.034
Grade 0-2	3 (6%)	11 (22%)	
Grade 3	47 (94%)	39 (78%)	

With regard to both immediately after stenting (p=0.022) and at the end of the procedure (p=0.018), the nicorandil group had a substantially larger number of patients who attained a TMPG of 3 than the control group (Table ). 

**Table 4:** Comparison of thrombolysis in myocardial infarction myocardial perfusion grade of the affected vessel among nicorandil and control groups Data is presented as n (%).

Thrombolysis in myocardial infarction myocardial perfusion grade	Nicorandil (n=50)	Control (n=50)	p-value
Immediately after stenting			0.022
Grade 0	2 (4%)	4 (8%)	
Grade 1	2 (4%)	5 (10%)	
Grade 0-2	9 (18%)	13 (26%)	
Grade 3	33 (66%)	20 (40%)	
End of procedure			0.018
Grade 0-2	4 (8%)	9 (18%)	
Grade 3	46 (92%)	41 (82%)	

The nicorandil group had a higher number of patients who had ST segment resolution than the control group (82% vs. 66%, p=0.008). Ventricular arrhythmias occurred significantly lower in the nicorandil group (4%) compared to the control group (16%) at 24 hours following pPCI (p=0.012). Six patients (12%) exhibited a transient reduction in blood pressure of 5-10 mmHg after receiving nicorandil, but they all recovered spontaneously.

## Discussion

The treatment impact of PCI in STEMI patients may be hampered by the complex pathophysiological process of STEMI, the presence of comorbidities, and consequences like NRP and myocardial reperfusion injury. As a result, further medication is needed to improve the patient's prognosis [2]. To the best of our knowledge, this was one of the very few studies conducted in India to find that the early administration of nicorandil distal to the coronary vascular lesion via a suction catheter prior to the incidence of no-reflow phenomenon decreased the occurrence of reperfusion injury, in-hospital cardiovascular mortality rates and re-hospitalizations rates than the controls in STEMI patients following pPCI.

The infusion of nicorandil via intracoronary and intravenous routes is widespread in STEMI patients. However, the efficacy of intracoronary administration of nicorandil over other forms for the prevention and treatment of NRP in STEMI patients is still debated [6]. The current investigation discovered that early intracoronary administration of nicorandil treatment was beneficial in decreasing NRP, and reperfusion arrhythmia, which was confirmed by a previous study done in 170 STEMI Chinese patients by Feng et al., 81 stable or unstable angina patients by Hwang et al. [7,8]. Similar to our study, Ezhilan et al. in 29 acute STEMI Indian patients, found that nicorandil prevents NRP in acute STEMI patients undergoing pPCI. Nevertheless, this previous study only compared clinical follow-up data at 12 months [9]. However, in addition to clinical follow-up at six months, our study compared the BNP, LVEDD, LVEF, TIMI 3 grade, and TMPG 3 grade among the nicorandil and control groups.

In the present study, though there was a non-significant difference found between the groups with regard to LVEF, the re-hospitalization rates were determined with the help of the following parameters: BNP, LVEDD for the assessment of the deterioration of congestive heart failure (CHF). We had found that nicorandil significantly improved BNP (348.45±112.32 pg/ml vs. 541.11±152.68 pg/ml, p=0.021) and LVEDD (54.12±3.56 mm vs. 60.62±4.98 mm, p=0.011) in STEMI patients undergoing primary PCI compared to the controls at 180^th^ day of admission. Similar to our study, Feng et al. found lower values of BNP (352.77±108.45 pg/ml vs. 542.31±155.77 pg/ml, p=0.039) and LVEDD (55.02±3.52 mm vs. 59.45±4.75 mm, p=0.021) in the nicorandil group compared to the control group [7]. Furthermore, the present study, in line with Feng et al. [7], found that the nicorandil group had a higher number of patients who attained TIMI 3 (p=0.034) and TMPG 3 (p=0.018) after pPCI. Owing to the aforementioned finding, the present investigation demonstrated that the use of nicorandil may improve cardiac systolic function and prognosis in STEMI patients who underwent pPCI by increasing myocardial blood flow reperfusion.

Numerous studies have suggested that the positive effect of nicorandil might be dose-dependent and revealed that the efficacy of intracoronary nicorandil to attenuate reperfusion injury was dependent on a wide range of follow-up times (range: 1, 3, and 6 months) and drug concentrations (dosage: 1, 2, 4, and 6 mg) [1,10-19]. The present study found that a dose of 2 mg of intracoronary nicorandil improved the coronary microcirculation than controls at a six-month follow-up, which is in accordance with the result of Feng et al. [7]. Similarly, Hwang et al. [8] discovered the efficacy of 2 mg intracoronary nicorandil prior to coronary angiography and 2 mg intracoronary prior to stent implantation at six months follow-up. Furthermore, Zhou et al., in a meta-analysis of 2965 STEMI patients, found that the use of nicorandil ranging from 2 to 6 mg bolus or 1.67 to 8mg/hour continuously for 3 to 24 hours is effective and does not result in adverse consequences [2]. Further long-term follow-up studies to determine the nicorandil treatment are warranted.

According to de Lemos et al. and Tomaszuk-Kazberuk et al., the prognosis following pPCI considerably varied between patients with and without rapid normalization of the ST segment (≥50% decline in ST-elevation) [20-21]. In line with these studies, Zhou et al. have demonstrated that the drop in amplitude of ST segment immediately following pPCI may represent the amount of reperfusion in the infarct-related myocardium [2]. Thus, the more noticeable the drop in amplitude of ST segment, the larger the blood flow to the damaged area and better the clinical prognosis. The present study found that 82% of patients in the nicorandil group showed a rapid decrease in ST segment deviation after pPCI, thus highlighting the beneficial effect of nicorandil in the decline of ST segment deviation.

The present study has a few drawbacks that need to be addressed. To begin with, the robustness of the current study findings may be influenced by the limited sample size and single-center study. In addition, observer bias or outcome reporting bias cannot be excluded from the current study due to the incidence of worsening heart failure. Furthermore, because nicorandil was compared to placebo rather than other medications, a head-to-head comparison with other drugs is needed to further demonstrate nicorandil's efficacy. Further multicentre, randomized controlled studies with large sample sizes are warranted.

## Conclusions

Early intracoronary administration of nicorandil as an adjuvant to pPCI in STEMI patients reduced the occurrence of NRP, in-hospital cardiovascular mortality rates, and re-hospitalization rates at six months following pPCI. Furthermore, it improved BNP and LVEDD and led to a higher number of TIMI flow grade 3 at the end of the procedure, TMPG flow grade 3, and ST segment elevation resolution, as well as reduced the incidence of reperfusion arrhythmias following pPCI. Larger studies with randomization are needed to confirm the beneficial effects of intracoronary nicorandil. Thus, nicorandil could be used as an adjunct to thrombosuction or intracoronary NTG during pPCI.
